# Ginseng Gintonin Enhances Hyaluronic Acid and Collagen Release from Human Dermal Fibroblasts Through Lysophosphatidic Acid Receptor Interaction

**DOI:** 10.3390/molecules24244438

**Published:** 2019-12-04

**Authors:** Rami Lee, Na-Eun Lee, Hongik Hwang, Hyewhon Rhim, Ik-Hyun Cho, Seung-Yeol Nah

**Affiliations:** 1 Ginsentology Research Laboratory and Department of Physiology, College of Veterinary Medicine, Konkuk University, Seoul 05029, Korea; rmlee12@konkuk.ac.kr (R.L.); dhkdnrl@naver.com (N.-E.L.); 2 Center for Neuroscience, Korea Institute of Science and Technology, Seoul 02792, Korea; hongik@kist.re.kr (H.H.); hrhim@kist.re.kr (H.R.); 3 Department of Convergence Medical Science, Department of Science in Korean Medicine and Brain Korea 21 Plus Program, Graduate School, Kyung Hee University, Seoul 02447, Korea; ihcho@khu.ac.kr

**Keywords:** ginseng, gintonin, human dermal fibroblast, hyaluronic acid, collagen, human skin

## Abstract

Gintonin is a newly discovered component of ginseng and acts as a ligand for G protein-coupled lysophosphatidic acid (LPA) receptors. It is currently unclear whether gintonin has skin-related effects. Here, we examined the effects of a gintonin-enriched fraction (GEF) on [Ca^2+^]_i_ transient induction in human dermal fibroblasts (HDFs). We found that GEF treatment transiently induced [Ca^2+^]_i_ in a dose-dependent manner. GEF also increased cell viability and proliferation, which could be blocked by Ki16425, an LPA1/3 receptor antagonist, or 1,2-Bis(2-aminophenoxy)ethane-*N*,*N*,*N*′,*N*′-tetraacetic acid tetrakis(acetoxymethyl ester) (BAPTA-AM), a calcium chelator. We further found that GEF stimulated hyaluronic acid (HA) release from HDFs in a dose- and time-dependent manner, which could be attenuated by Ki16425, U73122, a phospholipase C inhibitor, 2-Aminoethoxydiphenyl borate (2-APB), an IP_3_ receptor antagonist, and BAPTA-AM. Moreover, we found that GEF increased HA synthase 1 (HAS1) expression in a time-dependent manner. We also found that GEF stimulates collagen release and the expression of collagen 1, 3, and 7 synthases in a time-dependent manner. GEF-mediated collagen synthesis could be blocked by Ki16425, U73122, 2-APB, and BAPTA-AM. GEF treatment also increased the mRNA levels of LPA1-6 receptor subtypes at 8 h and increased the protein levels of LPA1-6 receptor subtypes at 8 h. Overall, these results indicate that the GEF-mediated transient induction of [Ca^2+^]_i_ is coupled to HA and collagen release from HDFs via LPA receptor regulations. We can, thus, conclude that GEF might exert a beneficial effect on human skin physiology via LPA receptors.

## 1. Introduction

Ginseng is a well-known traditional herbal medicine that has been used for its pharmacological and therapeutic effects in Korea, China, and Japan for thousands of years [1,2]. It has been studied extensively and has been shown to have effects on neurological disorders, such as Alzheimer’s disease (AD), stroke, Parkinson’s disease, and multiple sclerosis [3,4,5,6,7]. There have been several researches on ginseng itself, as well as on individual ingredients in ginseng, such as ginsenosides (a group of active ingredients in ginseng), most of which have focused on Rb1, Rg1, Rg3, Re, and Rd [8,9]. However, many studies on the pharmacological actions of ginseng have been limited to ginsenosides, even though ginsenosides alone cannot explain the wide variety of ginseng effects [7,10].

Gintonin, a component of ginseng and a novel exogenous ligand of G protein-coupled lysophosphatidic acid (LPA) receptors, has been identified as a non-saponin and non-acidic carbohydrate polymer [11,12]. The gintonin-enriched fraction (GEF) of ginseng extract contains a large amount of gintonin, as the name implies, and also linoleic acid (C18:2), phosphatidic acid (PA) 16:0-18:2, LPA C18:2, as well as other bioactive lysophospholipids in addition to LPAs [12]. GEF transiently induces [Ca^2+^]_i_ and stimulates diverse Ca^2+^-dependent cellular effects, ranging from neurotransmitter release to neuronal cell proliferation, through G protein-coupled LPA receptors [11,13]. Although the relationship between gintonin and the nervous system has been well-investigated, relatively little is known about the effects of gintonin on skin, specifically on fibroblasts that are closely related to skin biology.

Fibroblasts are one of the most important skin cell types, not only for proliferating, but also for maintaining skin homeostasis. LPA has been known to induce the proliferation and migration of fibroblasts [14]. Therefore, we hypothesized that GEF could be useful for revitalizing the skin by inducing the secretion of skin-hydrating or -protecting materials by stimulating fibroblast activities. Two major and well-known factors affecting skin hydration and protection are hyaluronic acid (HA) and collagen. HA is an extracellular matrix (ECM) component that hydrates skin, lubricates joints, and has a space-filling capacity. HA synthesis increases during tissue injury and wound healing. HA also regulates the activation of inflammatory cells to induce the cellular immune response upon fibroblast cell injury [14]. Collagen is required for deposition in fibroblasts and tissue regeneration [15,16]. *Panax* ginseng extract has wound-healing properties and has, thus, been used to maximize collagen synthesis for anti-wrinkle effects; however, its active ingredient(s) have not been clearly identified [17]. A large amount of research is, therefore, still needed to understand the effects of GEF binding to LPA receptors, as well as the relationship between GEF and skin biology.

In this study, we investigated how GEF promotes HA and collagen release. We found that GEF-mediated LPA receptor activation is coupled to Ca^2+^ signaling and is followed by HA and collagen release, which are closely associated with skin aging and skin protection. Thus, GEF can potentially be used as a cosmetic ingredient.

## 2. Results

### 2.1. GEF Induces [Ca^2+^]_i_ Transiently in Human Dermal Fibroblasts (HDFs)

**Figure 1:**
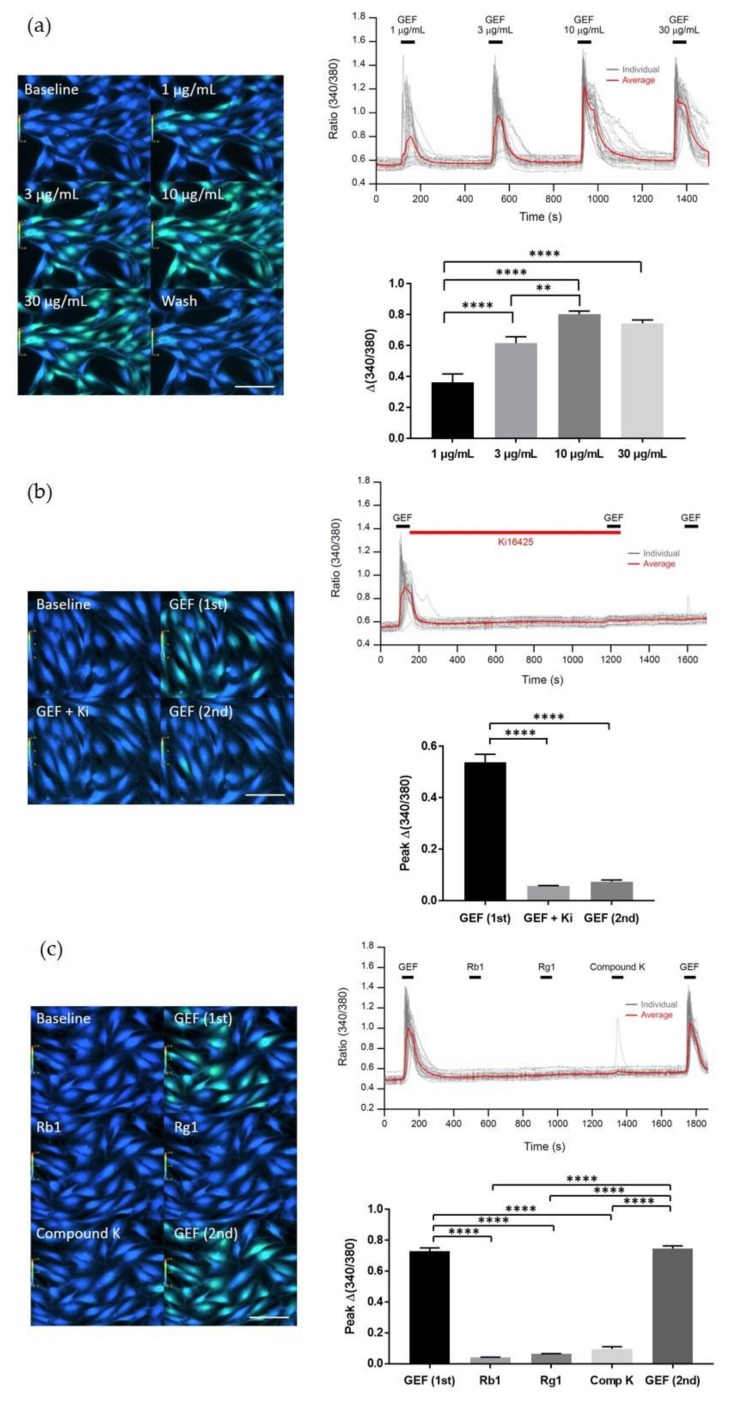
Transient induction of [Ca^2+^]_i_ in the absence or presence of gintonin-enriched fraction (GEF) or various ginsenosides. (**a**) GEF-mediated transient induction of [Ca^2+^]_i_ in human dermal fibroblasts (HDFs). HDFs were treated with GEF (1, 3, 10, or 30 μg/mL). GEF-mediated transient induction of [Ca^2+^]_i_ was dose-dependent. (**b**) The GEF-mediated [Ca^2+^]_i_ transient is blocked by Ki16425 in HDFs, (**c**) GEF but not ginsenosides such as ginsenosides (Rb1 and Rg1), or compound K induces [Ca^2+^]_i_ transient in HDFs. The data were obtained from 50–60 different cells in three independent experiments. The data are represented as the mean ±S.E.M. ** *p* < 0.01, **** *p* < 0.0001, one-way ANOVA followed by Tukey’s multiple comparisons test. Scale bar = 100 μm.

In previous reports, we showed that GEF induces the transient elevation of cytosolic Ca^2+^ levels during various cellular events, including neuronal and non-neuronal cell proliferation [2]. Here, based on these studies, we first examined whether treating HDFs with GEF transiently induces [Ca^2+^]_i_. As shown in Figure 1a, GEF treatment elevated [Ca^2+^]_i_ transiently, and this induction occurred in a concentration-dependent manner with the GEF concentration ranging from 1 to 10 µg/mL; the induction stopped at concentrations above 30 µg/mL of GEF. GEF-mediated transient induction of [Ca^2+^]_i_ was blocked by Ki16425, a LPA1/3 receptor antagonist, indicating the involvement of LPA1/3 receptors in this process (Figure 1b). However, ginsenoside Rb1, Rg1, and compound K had no effect on the transient induction of [Ca^2+^]_i_ by GEF in HDFs, indicating that GEF, but not ginsenosides, are the main cause of the transient induction of [Ca^2+^]_i_ via LPA receptor activation in non-neuronal HDFs (Figure 1c).

### 2.2. Effects of GEF on Cell Viability and Proliferation of HDFs

**Figure 2:**
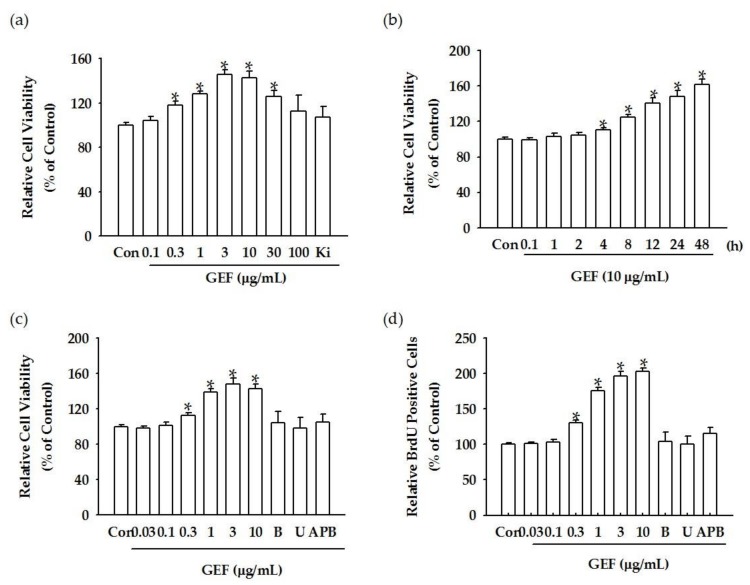
Effects of GEF on cell viability and proliferation in HDFs. (**a**) A concentration-dependent cell viability assay under GEF and Ki16425 treatment. Cells were treated with the indicated concentration of GEF for 24 h. Histograms for viability of HDFs treated with 0.1–100 μg/mL GEF (* *p* < 0.05) compared to that of HDFs treated with DMSO-treated vehicle control. Human dermal fibroblast viability was estimated using the WST-1 assay. * *p* < 0.05, control vs. GEF (0.3, 1, 3, 10, and 30 μg/mL) or Ki16425 (10 μM) + GEF (3 μg/mL) treatment. The data are represented as the mean ± S.E.M. (*n* = 3). (**b**) Time-course treatment effect of GEF on cell viability. Histograms show the time course of relative cell viability following treatment with 10 μg/mL GEF (* *p* < 0.05) compared to the control (0 h). Data are represented as the mean ± S.E.M. (*n* = 3). (**c**) GEF-mediated HDF cell viability was blocked by BAPTA-AM, U73122, and 2-APB. B: GEF + BAPTA-AM (10 μM); U: GEF + U73122 (5 μM); APB: GEF + 2-APB (50 μM). Histograms show the time course of relative cell viability following treatment with 10 μg/mL GEF (* *p* < 0.05) compared to the control (0 h) (*n* = 3). (**d**) GEF-mediated HDF BrdU incorporation is blocked by BAPTA-AM, U73122, and 2-APB. B: GEF + BAPTA (10 μM); U: GEF + U73122 (5 μM); 2-APB: GEF + 2-APB (50 μM). Histograms show the time course of relative cell viability following treatment with 10 μg/mL GEF (*n* = 3) (* *p* < 0.05) compared to the control (0 h).

Because the transient increase of cytosolic Ca^2+^ levels is closely related to cell proliferation [3], we examined the effects of GEF-mediated cell viability on HDF growth and proliferation. For this, we performed WST-1 and BrdU assays, which assess cell viability and proliferation as a function of cell number based on metabolic activity. As shown in Figure 2a,b, treatment of HDFs with GEF increased cell viability in a concentration- and a time-dependent manner in the WST-1 assay. Interestingly, GEF-mediated cell viability was attenuated by the LPA1/3 receptor antagonist Ki16425, by U73122, and by BAPTA-AM, an intracellular calcium chelator, implying that GEF-mediated cell proliferation involves the LPA1/3 receptor–Ca^2+^ signaling pathway (Figure 2c). GEF also increased BrdU incorporation, and GEF-mediated BrdU incorporation was blocked upon treatment of cells with U73122, 2-APB, or BAPTA-AM (Figure 2d). These results indicate that GEF-mediated transient induction of [Ca^2+^]_i_ is coupled with cell proliferation via GEF interaction with LPA receptors.

### 2.3. GEF Promotes Hyaluronic Acid (HA) Release and Affects HDFs Via LPA Receptors

**Figure 3:**
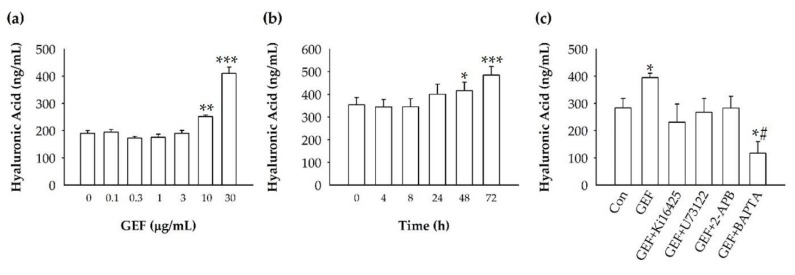
GEF-mediated hyaluronic acid release and inhibitory effect in HDFs. (**a**) Hyaluronic acid concentrations as ng/mL using the HA-ELISA kit. HDFs were treated with 0, 0.1, 0.3, 1, 3, 10, and 30 μg/mL GEF. The supernatant samples were collected after 48 h and their absorbance was measured using the HA-ELISA kit (Echelon) at a 405 nm wavelength. ** *p* < 0.01, *** *p* < 0.005. (**b**) Hyaluronic acid concentrations as ng/mL using the HA-ELISA kit. HDFs were treated with 10 μg/mL GEF and supernatant samples were collected after 4, 8, 24, 48, and 72 h and their absorbance was measured using the HA-ELISA kit (Echelon) at a 405 nm wavelength. * *p* < 0.05, *** *p* < 0.005. (**c**) Hyaluronic acid concentrations as ng/mL measured using the HA-ELISA kit. HDFs were treated with GEF (10 μg/mL), GEF + 2-APB (50 μM), GEF + U73122 (5 μM), GEF + Ki16425 (10 μM), or GEF + BAPTA-AM (10 μM); the supernatant samples were collected after 72 h and their absorbance was measured using the HA-ELISA kit (Echelon) at a 405 nm wavelength. * *p* < 0.05 vs. control, # *p* < 0.05 vs. GEF.

**Figure 4:**
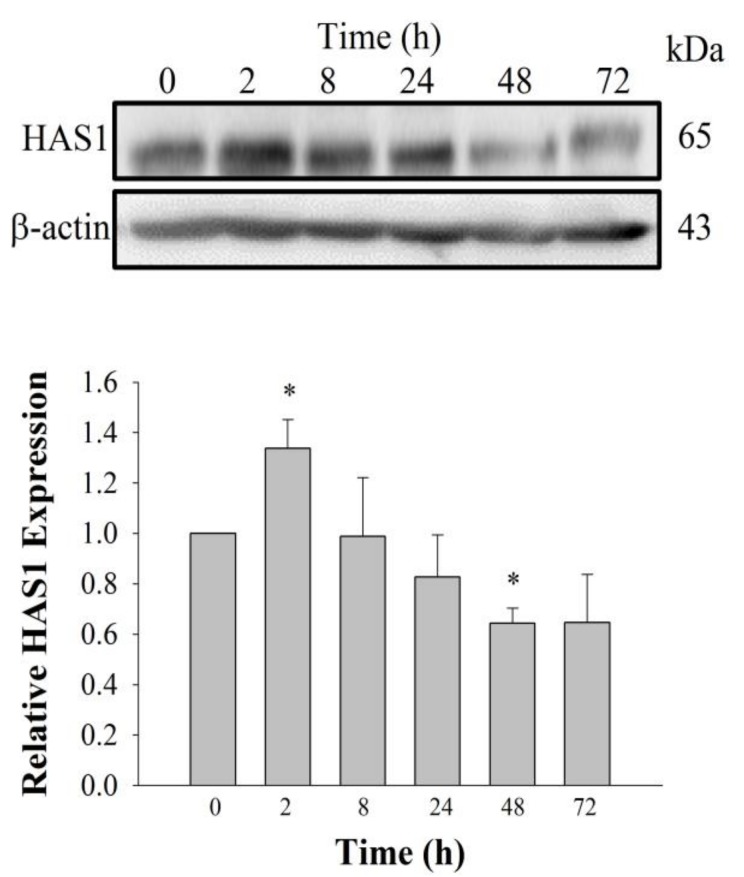
Time-dependent HAS1 expression changes. HDFs were treated with GEF (10 μg/mL) and HAS1 levels were measured by immunoblotting at 0, 2, 8, 24, 48, and 72 h. HAS1 expression increased at 2 h and, then, gradually decreased from 8 to 72 h. Data are represented as mean ± S.E.M. (*n* = 5). * *p* < 0.05 vs. control (0 h). One-way ANOVA was used to test statistical significance. All experiments were repeated six times (*n* = 6).

To understand the skin physiology-related effects of GEF on HDFs, we examined whether the amount of HA in the HDF growth media changed in response to GEF treatment using an HA-ELISA assay in the absence or presence of GEF. We treated HDFs with various concentrations of GEF (0, 0.1, 0.3, 1, 3, 10, or 30 μg/mL) and measured the amount of HA released into the media. GEF treatment increased the HA amount in the media in a dose-dependent manner, with significant increases observed at 10 and 30 μg/mL GEF (Figure 3a). To understand whether GEF-mediated HA release is time-dependent or not, we treated HDFs with 10 μg/mL GEF for different time periods (0, 4, 8, 24, 48, or 72 h). As shown in Figure 3b, HA release was the maximum at 72 h. Thus, HA release induced by GEF was time-dependent. Next, we examined the involvement of endogenous LPA receptors and Ca^2+^ in the HA release induced by GEF. Treatment with the LPA1/3 receptor antagonist Ki16425 (10 μM), the active phospholipase C inhibitor U73122 (5 μM), and inositol 1,4,5-trisphosphate receptor antagonist 2-APB (50 μM) attenuated GEF-mediated HA release by HDF cells (Figure 3c). Treatment with an intracellular Ca^2+^ chelator (BAPTA-AM, 10 μM) also blocked GEF-mediated HA release (Figure 3c). These results show that GEF induces HA release by HDF cells via the activation of the LPA receptor phospholipase C–intracellular IP_3_ receptor–Ca^2+^ signaling pathway. Next, we examined changes in HA synthase (HAS1) expression in HDFs after GEF treatment. GEF (10 μg/mL) treatment in HDFs increased HAS1 levels in a time-dependent manner. GEF treatment caused significant overexpression of HAS1 at 2 h compared to the expression of HAS1 at 0 h (control), and caused a decrease in HAS1 expression at 48 h, suggesting that GEF treatment of HDFs stimulates HAS1 expression only for a short time (Figure 4). We further tested whether Ki16425 affects GEF-induced HAS1 expression or not. We found that Ki16425 also blocked the effect of GEF on HAS1 protein expression (Figure S1).

### 2.4. Effects of GEF on Collagen Release in HDFs

**Figure 5:**
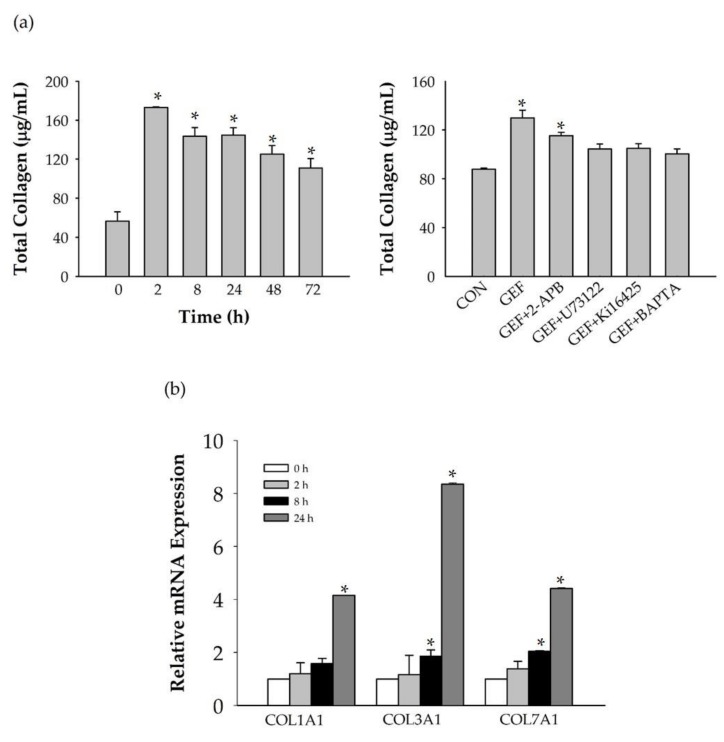
GEF-mediated total collagen release and inhibitory effects in HDFs. (**a**) GEF-mediated collagen release in HDF culture media. HDFs were treated with 10 μg/mL GEF; serum-free supernatant samples were collected after 2, 8, 24, 48, and 72 h and their absorbance was measured using the QuickZyme Total Collagen assay kit (Biosciences) at a 570 nm wavelength. * *p* < 0.05 vs. 0 h. (**b**) Total collagen as a μg/mL of the control using the HA-ELISA kit. HDFs were treated with GEF (10 μg/mL), GEF + 2-APB (50 μM), GEF + U73122 (5 μM), GEF + Ki16425 (10 μM), or GEF + BAPTA-AM (10 μM); the supernatant samples were collected after 24 h and their absorbance was measured using the QuickZyme Total Collagen assay kit (Biosciences) at a 570 nm wavelength. * *p* < 0.05 vs. control. (**b**) Time-dependent changes in collagen synthases’ mRNA expression levels. HDFs were treated with GEF (10 μg/mL). Data are expressed as the mean ± S.E.M. of triplicate samples analyzed using the 2^−ΔΔ^Ct method, with data normalized to the expression levels of the housekeeping gene β-actin. * *p* < 0.05 vs. control (0 h). For ELISA assay, all experiments were repeated five times (*n* = 5), and for mRNA analysis, the experiments were repeated three times (*n* = 3).

We further investigated the effect of GEF treatment on collagen release in HDFs for treatment times ranging from 0 to 72 h (Figure 5a). Cell culture media samples were collected and total collagen in the media was measured. GEF-mediated total collagen release into the media showed an increase at 2 h (Figure 5a, left panel). Longer GEF exposure times (8, 24, 48, and 72 h) also caused a significant increase in total collagen released into the media compared to the control (0 h) (Figure 5a). We also investigated the signaling pathways involved in collagen secretion by HDFs treated with GEF. GEF treatment alone increased collagen release significantly in HDFs. On the contrary, combining GEF treatment with U73122, Ki16425, 2-APB, or BAPTA-AM treatment inhibited the effect of GEF treatment after 24 h (Figure 5a, right panel).

Next, we examined changes in collagen synthase gene expression after GEF treatment in HDFs. To test the effects of GEF on collagen synthases, HDF was treated with GEF (10 μg/mL) for 2, 8, and 24 h. We found that GEF treatment caused a significant increase in collagen synthase expression (COL1A1, COL3A1, and COL7A1) in a time-dependent manner and caused significant overexpression at 24 h compared to the control (0 h). COL3A1 and COL7A1 expression was significantly upregulated at 8 h, suggesting that changes in collagen synthase gene expression are induced after a short time and are maintained in GEF-treated HDFs to promote collagen release (Figure 5b). We further tested the effect of Ki16425 on COL3A1 mRNA level and found that GEF-induced increase of COL3A1 mRNA levels was blocked (Figure S2).

### 2.5. Effects of GEF Treatment of HDFs on LPA Receptor Expression Level

**Figure 6:**
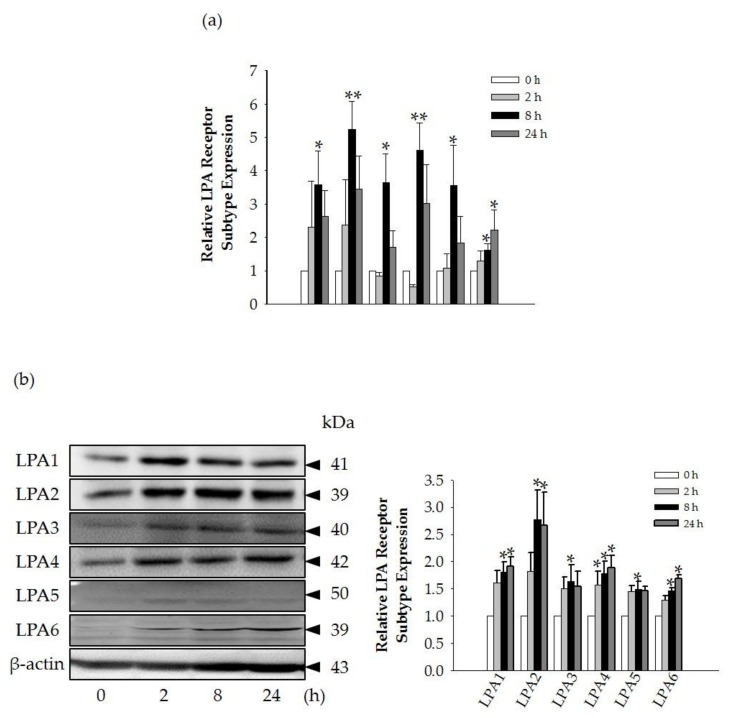
LPA receptor subtype expression changes in GEF-treated HDFs. (**a**) Time-dependent LPA receptor subtype mRNA expression changes. HDFs were treated with GEF (10 μg/mL). Data are expressed as the mean ± S.E.M. of triplicate samples of 2^−ΔΔ^Ct with data normalized to expression levels of the housekeeping gene β-actin. * *p* < 0.05 and ** *p* < 0.005 vs. control (0 h). (**b**) Time-dependent LPA receptor subtype protein expression changes. HDFs were treated with GEF (10 μg/mL). Data are expressed as the mean ± S.E.M. with data normalized to the expression levels of the housekeeping genes β-actin. * *p* < 0.05 vs. control. HDFs were treated with 10 μg/mL GEF for different periods of time (0, 2, 8, and 24 h). All experiments were repeated five times (*n* = 5).

Since GEF treatment stimulated HA and collagen release via the LPA receptor signaling pathway, we next investigated the changes in endogenous LPA receptor subtype expression levels in HDFs in the absence or presence of GEF. All LPA receptor subtype expression levels were significantly increased at 8 h compared to the control, while there were no significant changes at 2 and 24 h compared to the control (*p* < 0.05) (Figure 6a). The protein expression changes of LPA receptor subtypes in the presence of GEF exhibited a similar expression pattern to the mRNA expression changes. At 2 h, the protein levels of LPA1-6 receptors start to increased non-significantly, while protein expression levels of all LPA receptor subtypes were significantly increased at 8 h as shown in qRT-PCR analysis (Figure 6b). LPA1, 2, 4, and 6 exhibited significantly increased expression at 24 h. LPA3 and 5 also exhibited increased expression at 24 h.

## 3. Discussion

Skin is comprised of the epidermis and the dermis. The dermis is an inner layer that is comprised of fibroblasts. Skin acts as the front line of protection against bacterial infections, water loss, and physical injury [18,19,20]. It is well known that the fibroblast layer of skin is easily damaged by various factors, ranging from UV exposure to physical hazards [20]. Although previous reports have demonstrated that several ginsenosides and ginsenoside metabolites, such as compound K, exert skin-hydrating effects by regulating HA synthases and prevent skin aging and degradation of collagen by regulating collagenases under UVB irradiation conditions [20,21], other ingredients of ginseng, such as gintonin, have not been examined, and relatively little is known about how GEF affects the molecular mechanisms of skin homeostasis. To understand the mechanisms, we studied the beneficial effects of GEF on human skin fibroblasts.

We found that GEF treatment of HDF cells can induce [Ca^2+^]_i_ transiently via regulation of LPA receptor signal transduction and further induce in vitro cell proliferation via the LPA receptor signaling pathway. Interestingly, GEF, but not ginsenosides, such as ginsenoside Rb1, ginsenoside Rg1, and compound K, induced [Ca^2+^]_i_ transiently, indicating that GEF, but not ginsenosides, is an active ingredient of ginseng that promotes HDF cell regulation via transient induction of [Ca^2+^]_i_. Next, we examined whether GEF-mediated transient induction of [Ca^2+^]_i_ via LPA receptor regulation could be coupled to HA and collagen release via LPA receptor activation. We found that GEF stimulates HA and collagen release via the LPA receptor signaling pathway and that GEF treatment of HDF cells enhanced HA and collagen synthase gene expression. Finally, we found that GEF treatment increased LPA receptor subtype expression, at both the gene and protein levels. The present study, thus, shows that GEF-mediated cellular activity changes via LPA receptor subtype regulation are linked induction of [Ca^2+^]_i_, transient to cell proliferation, HA and collagen release, and changes in LPA receptor expression in HDF cells. These results are similar to those obtained in our previous studies on gintonin-mediated wound healing in corneal epithelium cells through in vitro, as well as in vivo experiments using a rabbit corneal injury model [22].

LPA, a lysophospholipid, acts as a ligand for at least six G protein-coupled LPA receptor subtypes (LPA1-6). LPA receptors are expressed in a variety of cells and tissues, including fibroblasts [14,15,22]. The present study provides further evidence that GEF treatment in normal human dermal fibroblast can help promote HA and collagen release by stimulating the LPA receptor–Ca^2+^ signaling pathway. Using cyclic phosphatidic acid, Maeda-Sano et al. revealed that HA synthesis takes place via the CREB transcription factor signaling pathway through the activation of HAS-2, and not HAS-1; this finding is not consistent with our results [15]. This discrepancy could be explained by our use of GEF, which is different from the compound used by Maeda-Sano et al. Moreover, we used a HAS-1 antibody, not HAS-2 antibody utilized by Maeda-Sano et al. to test protein expression levels (Figure 4) [15]. Further studies are needed to understand the genes and proteins that are responsible for induction of HA synthesis upon treatment of fibroblasts with GEF. These results also raise the possibility that GEF, an exogenous LPA receptor ligand, can play a key role in fibroblast hydration, as well as anti-aging if used as an externally applicable ingredient on the skin. Thus, GEF could be used as a base material for topical skin treatments. In the future, GEF could be used as an herb-derived cosmetic ingredient for daily skin treatment due to its skin-hydrating effect.

Interestingly, we found that the GEF-mediated transient induction of [Ca^2+^]_i_, cell proliferation, and HA and collagen release were attenuated by LPA1/3 receptor antagonists. However, as shown in Figure 6, we found that GEF treatment of HDFs increased the expression levels of nearly all of the six LPA receptor subtypes with different degrees in HDFs, indicating the possibility that GEF exerts its effects through all six LPA receptors. However, in the present study, we could not confirm the involvement of other LPA receptor subtypes except LPA1/3 receptors, due to the limited availability of selective and specific LPA receptor subtype antagonists. In future studies, the development of selective LPA receptor subtype antagonists will allow us to identify which LPA receptor subtypes are more involved in affecting HDF physiology with respect to HA and collagen release.

There was a report that matrix metallopeptidases (MMPs) may play an important role by LPA upregulating procollagen expression in normal skin conditions and downregulating MMP-1 under ultraviolet (UV)-induced conditions [23]. It is also known that UV irradiation causes up-regulation of several MMPs that can impair extracellular matrix (ECM) proteins that can lead to skin aging [24]. In future study, it will be interesting to examine the effects of GEF on MMPs related with skin biology.

In conclusion, we have demonstrated in this study that LPA receptor activation induced by GEF treatment of HDFs makes GEF an effective candidate for skin hydration and for possible anti-aging in HDFs. GEF is, therefore, a good candidate cosmetic ingredient to promote skin hydration, and possibly prevent or treat skin aging.

## 4. Materials and Methods

### 4.1. GEF Preparation and Reagents

GEFs were prepared as previously described [7]. Briefly, 4-year-old Korean white ginseng (Korea Ginseng Cooperation, Daejon, Korea), purchased locally from a ginseng market, was ground into small pieces (>3 mm) and refluxed with 70% fermentation ethanol. The ethanol extracts were concentrated and dissolved in distilled water. After ethanol extraction, the supernatant and precipitate produced by water fractionation were separated by centrifugation (1977× *g*, 20 min). The precipitate was lyophilized after being centrifuged. This fraction was designated GEF and had a yield of 1.3%. 2-Aminoethoxydiphenyl borate (2-APB), phospholipase C inhibitor (U73122), a membrane permeable Ca^2+^ chelator to chelate free cytosolic Ca^2+^ (calcium-specific aminoplolycarboxilic acid, BAPTA-AM), Ki16425 (LPA1/3 inhibitor), and other chemicals were purchased from Sigma-Aldrich (St. Louis, MO, USA). HAS1, LPA1-4, and anti-β-actin–HRP-conjugated antibodies were purchased from Abcam (Cambridge, MA, USA). LPA5 was obtained from Biorbyt (San Francisco, CA, USA), LPA6 was obtained from OriGene Technologies, Inc. (Rockville, MD, USA), and goat anti-rabbit IgG antibody was obtained from GeneTex (Irvine, CA, USA). For all molecular studies, the reagents and kits used were purchased from BIORAD (Hercules, CA, USA) and Invitrogen (Walthamm, MA, USA).

### 4.2. Cell Culture

For all experiments, human dermal fibroblasts (HDFs), from passages 3 to 10, were used (NB1RGB, Biosolution, Catalog No. MC1233). All cells were cultured in 95% Minimum Essential Medium (MEM) α with 5% fetal bovine serum and maintained at 37 °C in humidified conditions under 5% CO_2_. For testing dose dependence, cells were incubated in a 100-mm culture dish, followed by serum starvation for 24 h, and treated with vehicle or GEF (final concentration of 0, 1, 3, 10, 30, and 100 μg/mL for LPA subtype detection and cell viability test) for 24 h. Cells were incubated in a 100-mm culture dish, moved to fresh serum-free media for another 24 h, and treated with vehicle or GEF (final conc. of 10 μg/mL for HAS1 and LPA subtype detection and cell) for time dependence tests (0, 4, 8, 24, 48, and 72 h) or pre-treated with antagonists for 30 min followed by GEF treatment (final conc. of 10 μg/mL) for 24 h. Cell culture supernatants were used for detection of hyaluronic acid and collagen secretion into media.

### 4.3. Primer Designing and mRNA Analysis

To investigate mRNA expression of LPA receptors (LPA1-6), collagen type I, alpha 1 (*COL1A1*), collagen, type III, alpha 1 (*COL3A1*), and collagen, type VII, alpha 1 (*COL7A1*), β-actin expression was used as a normalization control in HDFs. Total RNA was isolated from HDFs with TRIzol (Invitrogen) according to the manufacturer’s instructions, with minor modifications. qRT-PCR (real-time PCR) was performed with individually reverse-transcribed cDNAs. The CFX96 real-time PCR system (BIORAD, U.S.A) was used for analysis. iQ SYBR Green Supermix (BIORAD) was utilized with the following steps: Pre-denaturation at 95 °C for 30 s, 35 repeats of denaturation at 95 °C for 10 s, 60 °C for 10 s, 72 °C for 25 s, final elongation at 72 °C for 30 s, and detection of melting curve from 72 °C to 97 °C. The following primers were used for this experiment: *LPA1*-5’gtcttctgggccattttcaa3´, 5´tcatagtcctctggcgaaca3´, 91 bp; *LPA2*-5´gaggccaactcactggtca3´, ggcgcatctcagcatctc3´, 58 bp; *LPA3*-5´gaagctaatgaagacggtgatga3´, 5´agcaggaaccaccttttcac3´, 135bp; *LPA4*-5´tctggatcctagtcctcagtgg3´, 5´ccagacacgtttggagaagc3´, 107 bp; *LPA5*-5´cgccatcttccagatgaac3´, 5´tagcggtccacgttgatg3´, 66 bp; *LPA6*-5´tctggcaattgtctacccatt3´, 5´tcaaagcaggcttctgagg3´, 165 bp; *β-actin*-5´ttctacaatgagctgcgtgtg3´, 5´ggggtgttgaaggtctcaaa3´, 122 bp; *COL1A1*-5´gccaagacgaagacatccca3´, 5´cagatcacgtcatcgcacaac3´, 136bp; *COL3A1*-5´cccactattattttggcacaacag3´, 5´aacggatcctgagtcacagaca3´, 129 bp; *COL7A1*-5´ctcagcagctatcacctggac3´, 5´tgtccaccacacgtagttcaa3´, 154 bp.

### 4.4. Immunoblotting

First, 20 μg of total protein from lysed HDF cells was used to detect LPA receptor subtypes and HAS1. The cells were lysed using modified radio immunoprecipitation assay buffer (RIPA buffer) with a protease inhibitor cocktail, and LPA receptor expression was detected using 10% sodium dodecyl sulfate polyacrylamide gel electrophoresis (SDS-PAGE) and semi-dry blotting of the protein onto a 0.45-μm PVDF membrane using rabbit anti-LPA1 polyclonal antibody (1:2000), rabbit anti-LPA2 polyclonal antibody (1:1000), rabbit anti-LPA3 polyclonal antibody (1:500), rabbit anti-LPA4 polyclonal antibody (1:2000), rabbit anti-LPA5 polyclonal antibody (1:2000), rabbit anti-LPA6 polyclonal antibody (1:2000), or rabbit anti-HAS1 polyclonal antibody (1:1000). The blotted membrane was stripped and re-probed with mouse anti-β-actin monoclonal antibody conjugated to HRP (1:30,000). Images were visualized using Clarity Western ECL Substrate Bio-Rad (Hercules, CA, USA) using the iBright CL1000 (Thermo Fischer Scientific, Waltham, MA, United States). Chemi-documentation was conducted for further documentation preservation. Densitometry analysis of the blots was performed using ImageJ.

### 4.5. Measuring Hyaluronic Acid Using Enzyme-Linked Immunosorbent Assay (ELISA)

Hyaluronic acid measurement was performed using the Hyaluronan Enzyme-Linked Immunosorbent Assay Kit (HA-ELISA, K-1201, Echelon, Salt Lake City, UT, USA) according to the manufacturer’s instructions. Briefly, cells were plated in a 100-mm dish (1 × 10^5^ cells/plate) for 24 h, serum-starved for another 24 h, then pre-treated with antagonists (2-APB, U73122, Ki16425, and BAPTA-AM) for 30 min, followed by GEF treatment. The collected supernatants were analyzed by the detection plate (K-1201, Echelon, Salt Lake City, UT, USA) and measured at 405 nm at T = 45 min using a scanning multi-well spectrophotometer.

### 4.6. Measuring Collagen Using Enzyme-Linked Immunosorbent Assay (ELISA)

Collagen analysis from cell media was conducted according to the manufacturer’s instructions. Briefly, cells were processed as described above, and the supernatants were collected, freeze-dried to yield powder, and re-suspended with 1× PBS (pH 7.2) to obtain 10× concentrated media. The samples were diluted at a 1:1 ratio with 12 M HCl, boiled at 95 °C for 20 h, and centrifuged for 10 min at 13,000× *g*. The prepared samples were treated with detection reagents for another 60 min and the plate was read at 570 nm by a scanning multi-well spectrophotometer (Spectra Max 190, Molecular Devices, Sunnyvale, CA, USA).

### 4.7. Cell Viability Assay

The cell viability after each treatment was determined using a water-soluble tetrazolium salt-1(WST-1) assay. The WST-1 cell proliferation assay (Catalog No. ab155902, Abcam, Cambridge, UK) was performed using a plate reader (Synergy 2, BioTek, Winooski, VT, USA). Cells were plated in 96-well plates (2 × 10^4^ cells/well) overnight and were subjected to GEF treatment for the indicated time. Then, the culture plate was incubated with the WST-1 reagent for 0.5–4 h, according to the manufacturer’s instructions. After incubation, the resulting formazan dye was quantified using a scanning multi-well spectrophotometer (Spectra Max 190, Molecular Devices, Sunnyvale, CA, USA). The optical density (OD) of each well was measured by a plate reader with a filter setting at 570 nm.

### 4.8. Intracellular Ca^2+^ Measurement

Intracellular Ca^2+^ levels in HDFs were measured after exposure to GEF. HDFs from each group were incubated for 40–60 min at room temperature with 5 μM Fura-2/AM (Molecular Probes, Eugene, OR, USA) and 0.001% pluronic F-127 (Molecular Probes) in a 4-(2-hydroxyethyl)-1-piperazineethanesulfonic acid (HEPES)-buffered solution composed of the following: 150 mM NaCl, 5 mM KCl, 1 mM MgCl_2_, 2 mM CaCl_2_, 10 mM HEPES, and 10 mM glucose, with the pH adjusted to 7.4 with NaOH. Cells were illuminated using a xenon arc lamp, and the excitation wavelengths (340 and 380 nm) were selected using a computer-controlled filter wheel (Sutter Instruments, Novato, CA, USA). The emitted fluorescence was reflected through a 515-nm-long pass filter to a frame transfer–cooled CCD camera (Olympus, Shinjuku, Tokyo, Japan), and the ratios of the emitted fluorescence were calculated using a digital fluorescence analyzer and then converted to intracellular free Ca^2+^ concentrations [Ca^2+^]_i_. All imaging data were collected and analyzed using the Universal Imaging software (Bedford Hills, New York, USA) [25].

### 4.9. Statistical Analysis

All values are presented as either mean ± standard error of the mean (S.E.M.) or % of control. A *p*-value under 0.05 was considered to be statistically significant. Differences between the means of the control and treatment groups were compared using unpaired Student’s *t*-test or one-way ANOVA.

## Supplementary Materials

The following are available online, Figure S1: The inhibitory effect of Ki16425 on GEF-induced HAS1 expression in HDFs, Figure S2: The inhibitory effect of Ki16425 on GEF-induced increase of COL3A1 gene expression in HDFs.
