# Supplementary material for: Ginseng Gintonin Enhances Hyaluronic Acid and Collagen Release from Human Dermal Fibroblasts Through Lysophosphatidic Acid Receptor Interaction
Source: Molecules. 2019 Dec 4;24(24):4438. doi: 10.3390/molecules24244438 (PMC6943507; doi:10.3390/molecules24244438)
Supplement: Supplementary file 1 [file molecules-24-04438-s001.pdf]

Supplementary Materials:

Ginseng gintonin enhances hyaluronic acid and collagen release from human dermal fibroblasts through lysophosphatidic acid receptor interaction

Rami Lee[1], Na-Eun Lee[1], Hongik Hwang[2], Hyewhon Rhim[2], Ik-Hyun Cho[3], and Seung-Yeol Nah[1,*]

[1] Ginsentology Research Laboratory and Department of Physiology, College of Veterinary Medicine, Konkuk University, Seoul 05029, Republic of Korea; rmlee12@konkuk.ac.kr, dhkdnrl@naver.com

[2] Center for Neuroscience, Korea Institute of Science and Technology, Seoul, 02792, Republic of Korea; e-hrhim@kist.re.kr, hongik@kist.re.kr

[3] Department of Convergence Medical Science, Department of Science in Korean Medicine and Brain Korea 21 Plus Program, Graduate School, Kyung Hee University, Seoul, 02447, Republic of Korea; ihcho@khu.ac.kr

*Correspondence: synah@konkuk.ac.kr; Tel.: +82-2-450-4154

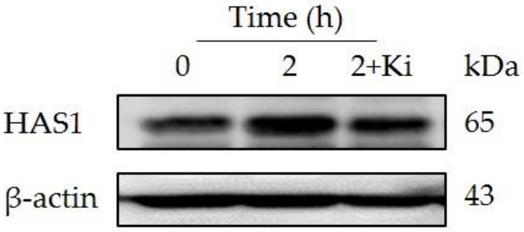

Figure S1. The inhibitory effect of Ki16425 on GEF-induced HAS1 expression in HDFs. HDFs were treated with GEF (10 μg/mL) and HAS1 levels were measured by immunoblotting at 0 and 2 h in the absence or presence of Ki16425 (10 μM). The increase of HAS1 expression by GEF was inhibited by Ki16425.

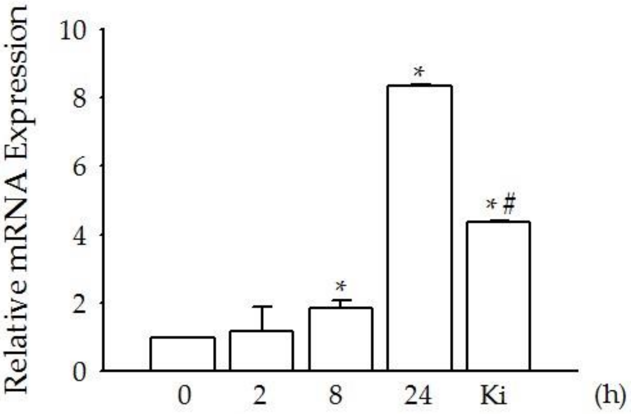

Figure S2. The inhibitory effect of Ki16425 on GEF-induced increase of COL3A1 gene expression in HDFs. GEF-mediated COL3A1 gene expression is blocked by Ki16425. HDFs were treated with GEF (10 μg/mL) for 0, 2, 8, and 24 h in the absence or presence of Ki16425 (10 μM, 24 h). Data are expressed as the mean ± S.E.M. of triplicate samples analyzed using the $2^{-\Delta\Delta}$Ct method, with data normalized to the expression levels of the housekeeping gene β–actin. *$P < 0.05$ vs. Control (0 and 24h); #$P < 0.05$ vs. 24 h. The experiments were repeated for 3 times (n=3).
